# Supplementary material for: Qubit gate operations in elliptically trapped polariton condensates
Source: Sci Rep. 2024 Feb 20;14:4211. doi: 10.1038/s41598-024-54543-6 (PMC10879284; doi:10.1038/s41598-024-54543-6)
Supplement: Supplementary file 1 — Supplementary Information. [file 41598_2024_54543_MOESM1_ESM.pdf]

SUPPLEMENTARY INFORMATION

Qubit Gate Operations in Elliptically Trapped Polariton Condensates

Luciano S. Ricco,[1,*] Ivan A. Shelykh,[1,2,3] and Alexey Kavokin[4,5,6,†]

[1]*Science Institute, University of Iceland,*
Dunhagi-3, IS-107 Reykjavik, Iceland

[2]*Russian Quantum Center, Skolkovo IC,*
Bolshoy Bulvar 30 bld. 1, Moscow 121205, Russia

[3]*Abrikosov Center for Theoretical Physics, MIPT,*
Dolgoprudnyi, Moscow Region 141707, Russia

[4]*Key Laboratory for Quantum Materials of Zhejiang Province,*
School of Science, Westlake University, 310024 Hangzhou, China

[5]*Institute of Natural Sciences, Westlake Institute for Advanced Study, 310024 Hangzhou, China*

[6]*Spin Optics Laboratory, St. Petersburg State University, 198504 St. Petersburg, Russia*

(Dated: January 19, 2024)

PAULI GATES

For completeness, below we rewrite the unitary operator given by Eq. (6) of the manuscript:

$$\hat{\mathcal{U}}(\mathcal{P}\tau) = \begin{bmatrix} \cos(\mathcal{P}\tau) - \imath\cos\phi\sin(|\mathcal{P}|\tau) & -\imath e^{-\imath\theta}\sin\phi\sin(\mathcal{P}\tau) \\ -\imath e^{\imath\theta}\sin\phi\sin(\mathcal{P}\tau) & \cos(\mathcal{P}\tau) + \imath\cos\phi\sin(\mathcal{P}\tau) \end{bmatrix}. \tag{1}$$

By considering the following parameters:

$$\hat{\mathcal{U}}\left(\tau = \frac{\pi}{2\mathcal{P}}, \theta = 0, \phi = \frac{\pi}{2}\right) = \begin{bmatrix} 0 & -\imath \\ -\imath & 0 \end{bmatrix} \equiv e^{-\imath\frac{\pi}{2}}\hat{X}_{\pi}, \tag{2}$$

$$\hat{\mathcal{U}}\left(\tau = \frac{\pi}{2\mathcal{P}}, \theta = \phi = \frac{\pi}{2}\right) = e^{-\imath\frac{\pi}{2}}\begin{bmatrix} 0 & -\imath \\ \imath & 0 \end{bmatrix} \equiv e^{-\imath\frac{\pi}{2}}\hat{Y}_{\pi}, \tag{3}$$

and

$$\hat{\mathcal{U}}\left(\tau = \frac{\pi}{2\mathcal{P}}, \theta = \forall, \phi = \pi\right) = \begin{bmatrix} \imath & 0 \\ 0 & -\imath \end{bmatrix} \equiv e^{\imath\frac{\pi}{2}}\hat{Z}_{\pi}, \tag{4}$$

the unitary operator corresponds to $\hat{X}_{\pi}$, $\hat{Y}_{\pi}$ and $\hat{Z}_{\pi}$ Pauli gates [1, 2], which rotates the qubit state by π radians around x-, y- and z-axis, respectively. Thus, by manipulating the auxiliary laser beam parameters, within a specified operational time τ, we can effectively implement all the Pauli gates. These single-qubit operations belong to a universal quantum gate set denoted as $\mathcal{G}_0 = \{\hat{X}_{\varphi}, \hat{Y}_{\varphi}, \hat{Z}_{\varphi}, \mathrm{Ph}_{\varphi}, \mathrm{CNOT}\}$ [2].

*i*SWAP GATE

Another kind of two-qubit operation that can be implemented in our elliptically trapped polariton condensate by means of tuning the parameters of the auxiliary laser beans and interaction between the traps is the so-called *i*SWAP gate [2]. This gate specifically requires an XY-type interaction between the qubits, i.e, $J_x = J_y = J_{12}$ and $J_z = 0$, which corresponds to

$$\hat{\mathcal{H}}_{\text{int}} = J_{12}(\hat{\sigma}_x \otimes \hat{\sigma}_x + \hat{\sigma}_y \otimes \hat{\sigma}_y). \tag{5}$$

By considering a trap of zero eccentricity so that $\Delta\varepsilon_j = 0$, and also a weak auxiliary laser beam $\mathcal{P}_x^{(j)}, \mathcal{P}_y^{(j)} \ll J_{12}$, the total two-qubit Hamiltonian [Eq. (9) in the manuscript] is reduced only to the Hamiltonian describing the XY-interaction in the qubit basis, cf. Eq. (5), with the corresponding

unitary time-evolution operator:

$$\hat{\mathcal{U}}_{12}(J_{1,2},\tau) = \begin{bmatrix} 1 & 0 & 0 & 0 \\ 0 & \cos(2J_{12}\tau) & -\imath\sin(2J_{12}\tau) & 0 \\ 0 & -\imath\sin(2J_{12}\tau) & \cos(2J_{12}\tau) & 0 \\ 0 & 0 & 0 & 1 \end{bmatrix}. \tag{6}$$

For $J_{12}\tau = \frac{\pi}{4}$, the unitary operator above corresponds exactly to the iSWAP gate as follows:

$$\hat{\mathcal{U}}_{12}\left(\frac{\pi}{4}\right) = \begin{bmatrix} 1 & 0 & 0 & 0 \\ 0 & 0 & -\imath & 0 \\ 0 & -\imath & 0 & 0 \\ 0 & 0 & 0 & 1 \end{bmatrix} \equiv i\text{SWAP}. \tag{7}$$

The iSWAP gate operation performs a state swap on the two-qubit system while introducing a phase difference of $\pi/2$. In practical terms, this means that in an illustrative scenario where the initial state is represented as $|\psi_0\rangle = |p_x\rangle_\mathrm{C}|p_y\rangle_\mathrm{T} \equiv |0\rangle_\mathrm{C}|1\rangle_\mathrm{T}$, the application of the iSWAP gate, as defined in Eq. (7), transforms it into $e^{-\imath\frac{\pi}{2}}|p_y\rangle_\mathrm{C}|p_x\rangle_\mathrm{T} \equiv e^{-\imath\frac{\pi}{2}}|1\rangle_\mathrm{C}|0\rangle_\mathrm{T}$ as the final state of the two-qubit system. Notice that both the CPHASE and iSWAP gates, as previously defined, need the condition $J_{12}\tau = \frac{\pi}{4}$, along with the presence of weak auxiliary laser beams. However, the distinction between performing the CPHASE and the iSWAP operation hinges on the adjustability of the ellipticity parameter $\Delta\varepsilon_j$.

* lsricco@hi.is

† a.kavokin@westlake.edu.cn

[1] M. A. Nielsen and I. L. Chuang, *Quantum Computation and Quantum Information: 10th Anniversary Edition* (Cambridge University Press, 2010).

[2] P. Krantz, M. Kjaergaard, F. Yan, T. P. Orlando, S. Gustavsson, and W. D. Oliver, A quantum engineer's guide to superconducting qubits, Applied Physics Reviews **6**, 021318 (2019).
